# Mortality in severe serious adverse events following heterologous and homologous prime-boost vaccination strategies for SARS-CoV-2: A retrospective cohort study

**DOI:** 10.1371/journal.pone.0323736

**Published:** 2025-05-23

**Authors:** Min Cheol Song, Jongmok Ha, Suyeon Park, Hyunwook Kang, Taeeun Kyung, Namoh Kim, Dong Kyu Kim, Kihoon Bae, Kwang Jun Lee, Euiho Lee, Jin Myoung Seok, Jinyoung Youn

**Affiliations:** 1 Infectious Disease Control Center, Gyeonggi Provincial Government, Suwon, Korea; 2 Department of Neurology, Samsung Medical Center, Sungkyunkwan University School of Medicine, Seoul, Korea; 3 Neuroscience Center, Samsung Medical Center, Seoul, Korea; 4 Department of Biostatistics, Academic Research Office, Soonchunhyang University Seoul Hospital, Seoul, Republic of Korea; 5 Department of Applied Statistics, Chung-Ang University, Seoul, Korea; 6 Department of Integrative Medicine, Yonsei University College of Medicine, Seoul, Korea; 7 Department of Internal Medicine, Yongin Severance Hospital, Yongin, Korea; 8 Department of Neurology, Soonchunhyang University Hospital Cheonan, Soonchunhyang University College of Medicine, Cheonan, Korea; Tecnológico de Monterrey, MEXICO

## Abstract

The COVID-19 pandemic underscored the urgent need for widespread vaccination to achieve herd immunity and mitigate severe outcomes. To address vaccine supply constraints, heterologous prime-boost strategies were adopted in Korea and other countries. Although studies have explored the effectiveness of heterologous prime-boost SARS-CoV-2 vaccination, comprehensive research on its adverse events (AEs), particularly severe serious AEs (SAEs), remains lacking. As an observational study, this study aims to compare severe SAEs across vaccination strategies and examine factors, including heterologous vaccination, associated with 42-day mortality among patients with severe SAEs, without implying causality. Our retrospective cohort study involved 358 cases of severe SAEs following prime-boost SARS-CoV-2 vaccination in Gyeonggi Province, South Korea, from February 26, 2021, to March 15, 2022. In patients with severe SAEs, the heterologous vaccination was associated with a higher risk of mortality than the homologous viral vector vaccination. Vaccinations performed at vaccination centers were associated with a lower risk of mortality. Furthermore, among patients with severe SAEs, the heterologous group exhibited a higher rate of respiratory diseases and genitourinary diseases compared to the homologous viral vector group. Moreover, the rate of deaths from genitourinary diseases among patients with severe SAEs was significantly higher in the heterologous group compared to the homologous viral vector group. We believe that our study, while limited to associations and not establishing causality, provides critical insights that could inform decision-making in scenarios where heterologous vaccination is necessitated by vaccine shortages or other constraints, particularly in managing severe SAEs and improving patient outcomes.

## Introduction

In the backdrop of the pandemic and the imperative to attain herd immunity while averting severe outcomes from the coronavirus disease 2019 (COVID-19), the need for substantial vaccination efforts became evident [1]. In 2021, South Korea authorized the use of four vaccines: mRNA-1273 (Moderna) and BNT162b2 (Pfizer-BioNTech) vaccines, which are mRNA-based, as well as Ad.26.COV2.S (Janssen) and ChAdOx1-S/nCoV-19 (Oxford-AstraZeneca) vaccines, which are viral vector-based [2]. By March 2022, 85.9% of the residents of Gyeonggi Province had received complete severe acute respiratory syndrome coronavirus 2 (SARS-CoV-2) vaccination.

Due to vaccine supply constraints, a heterologous prime-boost vaccination strategy for SARS-CoV-2 was implemented. This approach aimed to accelerate global vaccination efforts and enhance control of the pandemic [3]. In addition to South Korea, countries such as the USA, UK, and Sweden have adopted heterologous vaccine strategies, with relatively fewer issues related to vaccine supply. However, heterologous vaccines have been implemented in countries such as Bhutan as a solution to vaccine scarcity [4].

Recent systematic reviews and network meta-analyses have explored heterologous and homologous booster regimens, showing acceptable safety profiles and robust immunogenicity in adults [5–7]. Although studies have investigated adverse events (AEs) linked to heterologous vaccination [8,9], comprehensive research on this topic remains limited. In particular, data on the associations between various vaccination strategies, including heterologous regimens, and severe serious AEs (SAEs) are lacking.

Hence, the objectives of our study are to (1) assess differences in patient-specific and external factors among individuals who experience severe SAEs following SARS-CoV-2 vaccination, when categorized by vaccination strategies; (2) examine factors, including heterologous vaccination, associated with 42-day mortality among patients who experience severe SAEs; and (3) assess differences in the etiologies of severe SAEs across vaccination strategies and analyze the etiologies of deaths among severe SAEs across these strategies. We believe that our study provides critical insights that could inform decision-making in scenarios where heterologous vaccination is necessitated by vaccine shortages or other constraints.

## Methods

### Study design

We conducted a retrospective cohort study based on a community healthcare database analyzing physician- or self-reported severe SAEs temporally linked to SARS-CoV-2 vaccination in Gyeonggi Province, a prominent local governmental entity in South Korea inhabited by approximately 13 million people, representing almost one-third of the nation’s population, from February 26, 2021, to March 15, 2022. We examined differences in severe SAEs across vaccination strategies and evaluated the impact of specific strategies, including heterologous vaccination, on 42-day mortality among severe SAEs.

### Data collection

#### Passive surveillance on AEs following immunization.

A government-led passive surveillance program was established to monitor adverse events following immunization (AEFIs) caused by SARS-CoV-2 vaccination [2]. To encourage active participation, the Korean government provided expert feedback and monetary compensation for cases with a possible association between the vaccine and reported AEs. To address the limitations of passive surveillance, Gyeonggi Province implemented additional measures, including regular monitoring of reporting rates across all hospitals at the provincial level and distributing updated educational resources to in-hospital infection control centers to promote timely and accurate case reporting.

The Gyeonggi Infectious Disease Control Center (GIDCC) addressed all reported AEFI cases by thoroughly reviewing hospital electronic medical records and drug utilization review data provided by the Korean Health Insurance Review and Assessment Service. Additionally, the GIDCC conducted interviews with patients or their primary caregivers and discussed with relevant medical personnel to evaluate the cases.

#### Definition of cases.

The reported severe SAEs were defined as either deaths; events requiring intensive care unit (ICU) admission; life-threatening events; incidents resulting in permanent sequelae; or AEs of special interest (AESIs), such as anaphylaxis, thrombotic thrombocytopenia syndrome, myocarditis, pericarditis, and Guillain–Barré syndrome, which were temporally associated with SARS-CoV-2 vaccination.

Severe SAEs were defined as events that “result in significant morbidity or mortality,” encompassing ICU admissions, life-threatening conditions, severe damage, or deaths. For the sake of brevity, the term “severe SAEs” throughout the manuscript implies the occurrence of substantial morbidity or mortality.

The “homologous mRNA group” refers to patients with severe SAEs who exclusively received mRNA vaccines up to the second or third (booster) dose. The “homologous viral vector group” refers to patients with severe SAEs who exclusively received viral vector vaccines up to the second or third (booster) dose. The “heterologous group” represents patients with severe SAEs who received a viral vector vaccine followed by an mRNA vaccine up to the second or third (booster) dose. Notably, all individuals in the heterologous group in South Korea, including those in our study, received a viral vector vaccine followed by an mRNA vaccine.

#### Cohort and longitudinal follow-up.

Among the 38,828,691 administered SARS-CoV-2 vaccine doses, a total of 105,409 AEs were reported from February 26, 2021, to March 15, 2022. Among these, 687 patients were reported to have experienced severe SAEs temporally linked to SARS-CoV-2 vaccination. For the study, 23 cases were excluded as they did not meet the criteria of requiring ICU admission, being life-threatening, fatal, or resulting in long-term sequelae (N = 23). To facilitate a comparison between the homologous and heterologous groups, we excluded individuals who did not complete the vaccine series and received only one dose (N = 305) and an individual who received an inactivated vaccine (Sinopharm BBIBP-CorV) (N = 1). Among those who received two or more doses of the vaccine, 358 experienced severe SAEs. Initial follow-ups for all severe SAEs were carried out by the local community health center overseeing the participants’ residential district, followed by the GIDCC.

Consequently, the patients were categorized into three groups for survival analysis: the “homologous mRNA group” (N = 220), consisting of 90 deaths within 42 days of vaccination during the follow-up period; the “homologous viral vector group” (N = 75), consisting of 34 deaths within 42 days of vaccination during the follow-up period; and the “heterologous group” (N = 63), consisting of 36 deaths within 42 days of vaccination during the follow-up period.

**Fig 1:**
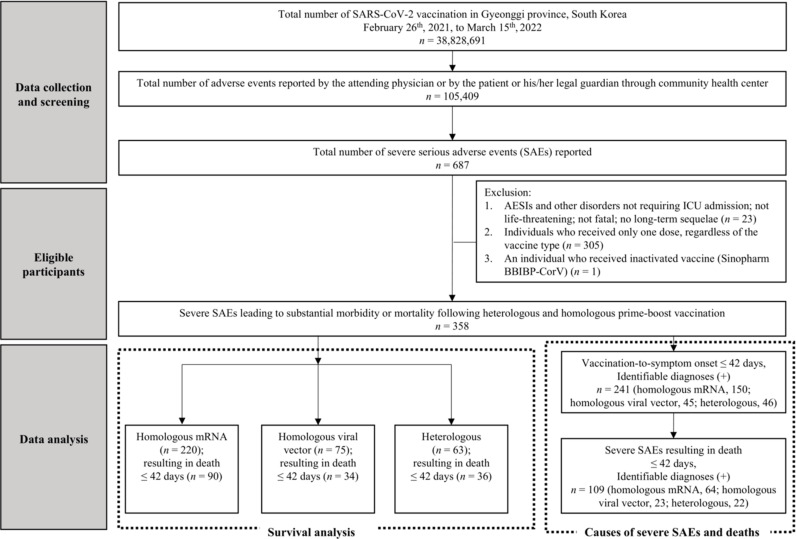
Flow chart of study participants and cohort selection. Abbreviations: SARS-CoV-2, severe acute respiratory syndrome coronavirus 2; SAEs, serious adverse events; AESIs, adverse events of special interest; ICU, intensive care unit.

To evaluate the etiologies of severe SAEs within 42 days of immunization across vaccination strategies, we ascertained 292 cases. Among them, 241 patients had identifiable diagnoses (homologous mRNA, N = 150; homologous viral vector, N = 45; heterologous, N = 46). Of the 160 severe SAE cases that led to mortality within 42 days, 109 were associated with identifiable diagnoses (homologous mRNA, N = 64; homologous viral vector, N = 23; heterologous, N = 22) (Fig 1).

#### Covariates.

We selected potential risk factors associated with mortality in severe SAEs following SARS-CoV-2 vaccination, based on clinical importance and findings from previously published studies [10,11]. These covariates included vaccination strategy (homologous mRNA, homologous viral vector, or heterologous), age, sex, number of doses received (two or three), vaccination site (medical institution, vaccination center, nursing hospital and facility, or community health center), and pertinent comorbidities.

Comorbidities were quantified using the Charlson Comorbidity Index (CCI), a validated scoring system predicting 10-year mortality in patients with multiple conditions. The CCI includes age as well as the following conditions: cardiovascular diseases (myocardial infarction, congestive heart failure, peripheral vascular disease), cerebrovascular diseases (stroke or transient ischemic attack), dementia, chronic respiratory diseases (chronic obstructive pulmonary disease or asthma), connective tissue diseases, peptic ulcer disease, liver disease, diabetes mellitus, neurological disabilities (paraplegia or hemiplegia), chronic kidney disease, solid tumors, hematologic malignancies (e.g., leukemia, lymphoma), and HIV/AIDS [12]. Individual comorbid conditions are detailed in Table 1. However, we used the CCI in our multivariable analysis as a standardized measure of overall comorbidity burden and did not evaluate the independent effects of each condition. For statistical analyses, the CCI scores were categorized into two groups using a cut-off value of 2: low (≤2) and high (>2) [13,14]. This index provided a standardized measure to assess the impact of comorbidity burden on mortality outcomes.

**Table 1:** Baseline characteristics of patients who encountered severe SAEs subsequent to SARS-CoV-2 vaccination, categorized by vaccination strategies.

	Total (N = 358)	Vaccination strategy			*P*
		Homo-mRNA (N = 220)	Homo-viral vector (N = 75)	Heterologous (N = 63)	
Age, median (IQR), y	69 (57, 79)	75 (53, 81)	67 (62, 72)	69 (62, 73)	0.206^a^
Sex, N (%)					0.062
Female	131 (36.6)	85 (38.6)	19 (25.3)	27 (42.9)	
Male	227 (63.4)	135 (61.4)	56 (74.7)	36 (57.1)	
Number of doses received, N (%)					<0.001
2	240 (67.0)	150 (68.2)	75 (100)	15 (23.8)	
3	118 (33.0)	70 (31.8)	0 (0)	48 (76.2)	
Vaccination site, N (%)^c^					<0.001^b^
Medical institution	245 (68.4)	124 (56.4)	63 (84.0)	58 (92.1)	
Vaccination center	93 (26.0)	93 (42.3)	0 (0)	0 (0)	
Nursing hospital and facility	10 (2.8)	3 (1.4)	4 (5.3)	3 (4.8)	
Community health center	10 (2.8)	0 (0)	8 (10.7)	2 (3.2)	
CCI, N (%)					0.465
≤ 2	120 (33.5)	79 (35.9)	23 (30.7)	18 (28.6)	
> 2	238 (66.5)	141 (64.1)	52 (69.3)	45 (71.4)	
Comorbidities, N (%)					
Hypertension	188 (52.5)	111 (50.5)	40 (53.3)	37 (58.7)	0.504
Diabetes mellitus	129 (36.0)	69 (31.4)	32 (42.7)	28 (44.4)	0.066
Dyslipidemia	87 (24.3)	49 (22.3)	23 (30.7)	15 (23.8)	0.341
Heart failure	8 (2.2)	6 (2.7)	0 (0)	2 (3.2)	0.406^b^
Arrhythmia	22 (6.1)	15 (6.8)	4 (5.3)	3 (4.8)	0.861^b^
Ischemic heart disease	38 (10.6)	25 (11.4)	4 (5.3)	9 (14.3)	0.199
Stroke	38 (10.6)	21 (9.5)	5 (6.7)	12 (19.0)	0.045
Epilepsy	7 (2.0)	3 (1.4)	2 (2.7)	2 (3.2)	0.487^b^
Parkinson’s disease	4 (1.1)	3 (1.4)	0 (0)	1 (1.6)	0.636^b^
Dementia	42 (11.7)	24 (10.9)	9 (12.0)	9 (14.3)	0.761
Psychiatric disorder	12 (3.4)	7 (3.2)	4 (5.3)	1 (1.6)	0.447^b^
Asthma	17 (4.7)	10 (4.5)	2 (2.7)	5 (7.9)	0.357^b^
Thyroid disorder	13 (3.6)	4 (1.8)	4 (5.3)	5 (7.9)	0.034^b^
Chronic liver disease	7 (2.0)	2 (0.9)	3 (4.0)	2 (3.2)	0.103^b^
Chronic kidney disease	20 (5.6)	9 (4.1)	3 (4.0)	8 (12.7)	0.048^b^
Benign prostatic hyperplasia	17 (4.7)	12 (5.5)	3 (4.0)	2 (3.2)	0.827^b^
Cancer	22 (6.1)	12 (5.5)	5 (6.7)	5 (7.9)	0.706^b^

Note: Age is displayed as median (interquartile range), while categorical variables are represented as N (%).

Abbreviations: SAEs, serious adverse events; SARS-CoV-2, severe acute respiratory syndrome coronavirus 2; Homo-mRNA, Homologous mRNA; Homo-viral vector, Homologous viral vector; IQR, interquartile range; CCI, Charlson Comorbidity Index.

^a^Kruskal-Wallis H test was employed for the analyses.

^b^Fisher’s exact test was employed for the analyses.

^c^The vaccination site with the nearest temporal proximity to the adverse event was selected for the analysis.

#### Diagnostic classification of severe SAEs.

As described in our previous study, primary clinical diagnoses were categorized using the International Classification of Diseases 10^th^ Revision (ICD-10) and ICD-10 Clinical Modification classification schemes and grouped into specific etiological categories [15]. In cases where mortality was reported as severe SAE with no clear diagnosis, it was classified as severe SAE without an identifiable diagnosis (S1 Text).

### Statistical analyses

We established descriptive statistics based on the data characteristics. We presented continuous data as medians and interquartile ranges (IQRs), whereas categorical data as absolute counts and relative frequencies (N [%]). We employed the Kruskal-Wallis test and the chi-square test or Fisher’s exact test for continuous and categorical variables, respectively (Table 1).

**Fig 2:**
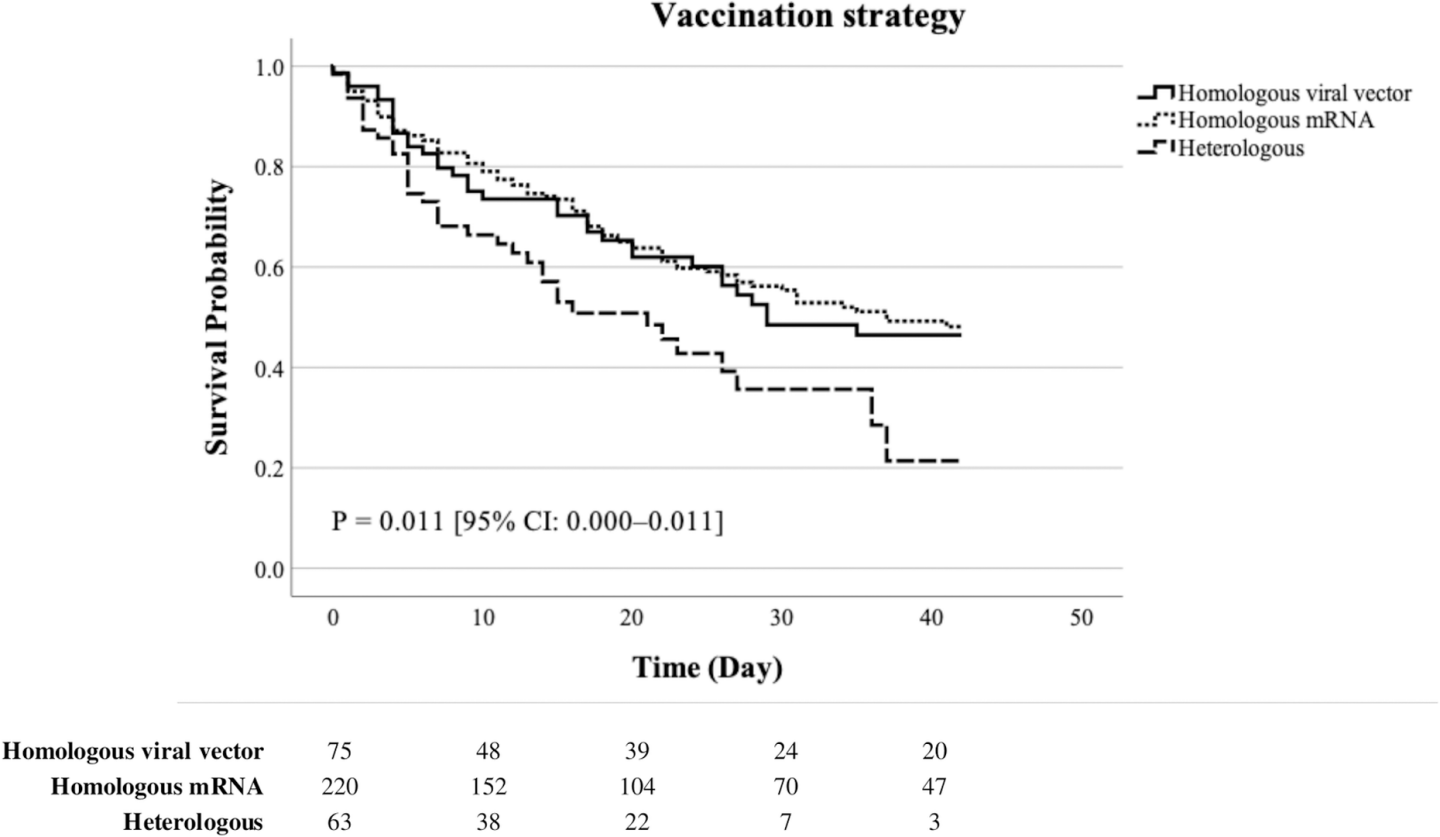
Kaplan–Meier survival curves for patients experiencing severe serious adverse events by vaccination strategies. Abbreviations: CI, confidence interval; SAEs, serious adverse events. Note: The ‘heterologous’ represents patients with severe SAEs who received a viral vector vaccine followed by an mRNA vaccine up to the second or third (booster) dose. The ‘homologous mRNA’ refers to patients with severe SAEs who exclusively received mRNA vaccines up to the second or third (booster) dose. The ‘homologous viral vector’ refers to patients with severe SAEs who exclusively received viral vector vaccines up to the second or third (booster) dose. The number at risk for each time interval is displayed below the x-axis.

Kaplan-Meier curves were used to visualize the difference in survival probability between the vaccination strategies in patients with severe SAEs during the 42-day follow-up period (Fig 2). Additionally, the mean survival time was calculated. Univariate and multivariate Cox proportional hazards (PH) regression analyses were conducted to examine the associations among vaccination strategy, sex, vaccination site, and CCI with mortality in patients experiencing severe SAEs (Table 2). Variable selection in multivariate analysis was performed using backward likelihood ratio selection. We tested the PH assumption for the Cox regression model using Schoenfeld residuals. As CCI violated the PH assumption (*p* = 0.003), we performed a multivariable Cox regression analysis with stratification by CCI.

**Table 2:** Factors associated with mortality in severe SAEs following SARS-CoV-2 vaccination: patient-specific and external factors.

		Univariable analysis			Multivariable analysis		
		HR	(95% CI)	*p-value*	aHR	(95% CI)	*p-value*
Vaccination strategy	Homologous viral vector	1.000			1.000		
	Homologous mRNA	0.936	(0.631-1.388)	0.741	1.509	(0.972-2.344)	0.067
	Heterologous	1.660	(1.036-2.659)	0.035	1.719	(1.064-2.775)	0.027
Sex	Female	1.000					
	Male	0.992	(0.720-1.368)	0.962			
Vaccination site^a^	Vaccination center	1.000			1.000		
	Medical institution	2.227	(1.438-3.449)	<0.001	2.776	(1.692-4.555)	<0.001
	Nursing hospital and facility	3.740	(1.611-8.685)	0.002	4.374	(1.816-10.535)	0.001
	Community health center	2.198	(0.761-6.348)	0.145	3.945	(1.234-12.614)	0.021
CCI	≤ 2	1.000					
	> 2	1.323	(0.945-1.851)	0.103			

Abbreviations: SAEs, serious adverse events; SARS-CoV-2, severe acute respiratory syndrome coronavirus 2; HR, hazard ratio; aHR, adjusted hazard ratio; CI, confidence interval; CCI, Charlson Comorbidity Index.

^a^The vaccination site with the nearest temporal proximity to the adverse event was selected for the analysis.

Multivariable analysis adjusted for vaccination strategy and vaccination site, stratified by CCI.

**Fig 3:**
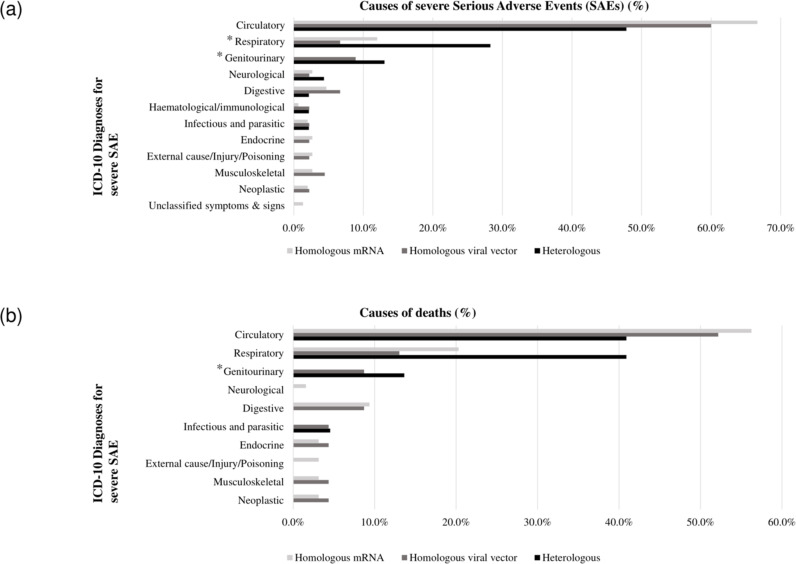
Rates of etiologies for severe serious adverse events and subsequent deaths by vaccination strategies. Abbreviations: SAEs, serious adverse events; ICD-10, International Classification of Diseases 10th Revision. Note: The asterisk (*) indicates ICD-10 diagnoses that exhibited a statistically significant difference among vaccination strategies. (a) Each bar represents the rate of causes of severe SAEs categorized by vaccination strategy, classified according to the etiological classification based on the ICD-10 diagnoses. (b) Each bar represents the rate of causes of subsequent deaths categorized by vaccination strategy, classified according to ICD-10 diagnoses.

The rate of specific causes of severe SAEs and subsequent deaths for each vaccination strategy was calculated by dividing the number of patients who experienced severe SAEs or deaths classified under ICD-10 by the total number of patients with confirmed severe SAEs or deaths and subsequently multiplying the result by 100 (Fig 3). Statistical analyses were performed using the IBM SPSS Statistics (version 29.0; IBM, Armonk, NY, USA) and the R statistical software program (version 4.3.1; R Core Team 2023). Statistical significance was set at 0.05.

### Ethics approval and consent to participate

This study was exempt from review by the Korean Public Institutional Review Board (identifier: P01-202204-01-006). This exemption was granted because it utilized de-identified data already collected through the epidemiological investigation for analysis, aligned with current public health interests and posed minimal risk to participants. Written informed consent was obtained from all participants or their legal guardians during the initial epidemiological investigation. For minors, consent was obtained from their parents or legal guardians. All methods adhered to the Strengthening the Reporting of Observational Studies in Epidemiology guidelines.

## Results

### Baseline characteristics of patients with severe SAEs following SARS-CoV-2 vaccination categorized by vaccination strategies

The median age of the 358 individuals who experienced severe SAEs was 69 years (IQR: 57–79 years). A total of 63.4% of the patients were male, and 33.0% had received a third dose of vaccination. Most patients were vaccinated at medical institutions (68.4%) or vaccination centers (26.0%). More patients had CCI higher than two (66.5%).

No significant age or sex differences were observed among the vaccination strategies. However, the number of doses administered before the onset of severe SAEs differed significantly. Most patients from the homologous mRNA and viral vector groups received two doses (68.2% and 100%, respectively), whereas most patients from the heterologous group received three doses (76.2%; *p* < 0.001). In addition, the homologous viral vector and heterologous groups were more likely to be vaccinated at medical institutions (84.0% and 92.1%, respectively), whereas only patients from the homologous mRNA group were vaccinated at vaccination centers (42.3%; *p* < 0.001). There was no significant difference in CCI between the groups. However, the heterologous group had a larger proportion of patients with stroke (19.0%; *p* *=* *0.045*), thyroid disorder (7.9%; *p* *=* *0.034*), and chronic kidney disease (12.7%; *p* *=* *0.048*) (Table 1).

### Patient-specific and external factors associated with mortality in patients with severe SAEs following SARS-CoV-2 vaccination

The overall mean survival time was 26.827 days (95% confidence interval [CI], 25.064–28.590). In the univariate analysis, neither sex nor CCI was significantly associated with mortality. However, in patients with severe SAEs, the heterologous group was associated with a higher risk of mortality compared to the homologous viral vector group (crude hazard ratio [cHR], 1.660; 95% CI, 1.036–2.659). Additionally, patients with severe SAEs who were vaccinated at medical institutions, and nursing hospitals and facilities were associated with a higher risk of mortality than those vaccinated at vaccination centers (cHR, 2.227 and 3.740; 95% CI, 1.438–3.449 and 1.611–8.685, respectively).

Consistently, the multivariate analysis revealed significant differences in vaccination strategy and site. In patients with severe SAEs, the heterologous group was associated with a higher risk of mortality compared to the homologous viral vector group (adjusted hazard ratio [aHR], 1.719; 95% CI, 1.064–2.775). Patients with severe SAEs who were vaccinated at medical institutions, nursing hospitals and facilities, and community health centers were associated with a higher risk of mortality than those vaccinated at vaccination centers (aHR, 2.776, 4.374, and 3.945; 95% CI, 1.692–4.555, 1.816–10.535, and 1.234–12.614, respectively) (Table 2, Fig 2).

### Specific causes of severe SAEs and subsequent deaths across vaccination strategies in patients with severe SAEs

In the homologous mRNA group, diseases of the circulatory, respiratory, and digestive systems (in sequential order) were the leading causes of severe SAEs (66.7%, 12.0%, and 4.7%, respectively) and deaths among patients with severe SAEs (56.3%, 20.3%, and 9.4%, respectively).

In the homologous viral vector group, circulatory, genitourinary, respiratory, and digestive system diseases were the leading causes of severe SAEs (60.0%, 8.9%, 6.7%, and 6.7%, respectively). The leading causes of deaths among patients with severe SAEs were related to the circulatory, respiratory, genitourinary, and digestive systems (52.2%, 13.0%, 8.7%, and 8.7%, respectively).

In the heterologous group, circulatory, respiratory, and genitourinary diseases were the leading causes of severe SAEs (47.8%, 28.3%, and 13.0%, respectively) and deaths among patients with severe SAEs (40.9%, 40.9%, and 13.6%, respectively).

In patients with severe SAEs, the heterologous group had a higher rate of respiratory diseases compared to the homologous viral vector group (28.3% and 6.7%, respectively; *p *= 0.006). Nevertheless, there were no significant differences in deaths due to respiratory diseases among patients with severe SAEs across the three vaccination strategies. Furthermore, in patients with severe SAEs, the heterologous group showed a higher rate of genitourinary diseases than the homologous viral vector group (13.0% and 8.9%, respectively; *p *< 0.001). Notably, none of the severe SAEs in the homologous mRNA group was associated with genitourinary diseases. Moreover, a significant difference in deaths due to genitourinary diseases among patients with severe SAEs was observed between the heterologous and homologous viral vector groups (13.6% and 8.7%, respectively; *p *= 0.007) (Fig 3).

## Discussion

We performed a retrospective cohort study of 358 severe SAE cases that resulted in significant morbidity or mortality following specific vaccination strategies. The main findings of this study include: among patients with severe SAEs, (1) the heterologous group was associated with a higher risk of mortality compared to the homologous viral vector group; (2) vaccinations performed at vaccination centers were associated with a lower risk of mortality; and (3) the heterologous group exhibited increased rates of severe SAEs involving respiratory and genitourinary diseases, as well as a higher rate of deaths related to genitourinary diseases among patients with severe SAEs. To our knowledge, this study is the first to examine the differences in severe SAEs across prime-boost vaccination strategies and evaluate the impact of specific strategies, including heterologous prime-boost vaccination, on 42-day mortality among severe SAEs.

Our findings indicate that the heterologous group was associated with poorer survival outcomes compared to the homologous viral vector group in patients with severe SAEs, though this does not imply causation. Notably, this finding was significant after adjusting for vaccination site and stratifying by CCI. Excessive and unintended immune responses, sometimes triggered by molecular mimicry, may be the primary cause of a significant proportion of AEFIs [16]. Hence, it is crucial to scrutinize immunogenicity variations among different vaccination strategies. Studies have shown that heterologous viral vector-primed mRNA vaccinations are more immunogenic than are homologous viral vector vaccinations [17]. In the heterologous viral vector-primed mRNA vaccination, both humoral response (as evidenced by SARS-CoV-2 anti-spike IgG concentrations) and cellular response (measured through IFNγ ELISpot) were notably elevated [18]. Furthermore, from a clinical perspective, higher systemic reactogenicity following the booster dose was observed with heterologous prime-boost vaccination than with homologous prime-boost vaccination [19]. Therefore, increased immunogenicity and reactogenicity could explain the higher mortality risk associated with the heterologous group.

Patients with severe SAEs vaccinated at vaccination centers were associated with better survival outcomes than those vaccinated at other sites. This difference may reflect the influence of preexisting health conditions on vaccination location. For example, during the early vaccination phase, mobile individuals aged 75 years and older were directed to vaccination centers, while residents of long-term care facilities received on-site vaccinations [20]. Because the preventive effect of vaccination may be consistent irrespective of underlying medical conditions [21], it is believed that vaccination remains necessary.

Finally, the heterologous group demonstrated an increased rate of severe SAEs associated with respiratory and genitourinary diseases, as well as a higher rate of deaths attributed to genitourinary diseases among patients with severe SAEs. Of the 34 severe SAEs with identifiable diagnoses associated with respiratory diseases, only four cases were not related to respiratory pneumonia. Exceptions included one case of acute epiglottitis in the homologous mRNA group and one case each of acute respiratory failure, hypersensitivity pneumonitis, and acute exacerbation of COPD in the heterologous group. In genitourinary diseases, among the 10 severe SAEs with identifiable diagnoses, all but one were urinary tract infections, except for acute renal failure in the homologous viral vector group. In these cases, all cases of mortality were due to urinary tract infection. Although caution is warranted owing to the absence of a clearly defined mechanism, we consider transient lymphopenia as a potential reason for the predominance of pneumonia and urinary tract infection. Transient lymphopenia was observed in a dose-dependent manner following mRNA vaccination [22]. Because lymphopenia is associated with an elevated risk of infection and subsequent risk of mortality [23], we hypothesize that this may account for the predominance of pneumonia and urinary tract infections in the heterologous group, which may have experienced higher levels of reactogenicity than the other groups.

This study has certain limitations that warrant acknowledgment. First, the study focuses exclusively on patients with severe SAEs, which may limit the generalizability of the findings to the general population. Caution is warranted when applying these results to broader groups, as the study population represents a specific subset with distinct characteristics. Second, in each vaccine strategy group, there is a mixture of patients who received either a second or third dose (booster). Specifically, in the heterologous viral vector-primed mRNA group, there is a combination of patients who received mRNA vaccination at either the second or third dose. This variability may contribute to differences in detailed immunogenicity. Third, because this study is purely observational, it identifies only associations between variables and does not establish any causal relationships. However, the temporal links identified in our study offer valuable insights that can guide future investigations into causation. Fourth, it is important to note that the increased mortality associated with the heterologous group may be partly attributable to factors other than the vaccination strategy itself, such as selection bias, reporting bias, and unmeasured confounders, including time interval between vaccine doses, socioeconomic status, healthcare access, and the severity of severe SAEs. Although our analysis adjusted for known confounders, residual confounding cannot be completely ruled out. Fifth, the number of deaths related to SARS-CoV-2 infection during the follow-up period was not fully accounted for, which may have influenced the interpretation of mortality outcomes. Nationwide time-series analyses exploring the relationships between SARS-CoV-2 infection, vaccination, and deaths following severe SAEs could help clarify these temporal associations. Lastly, passive surveillance might be affected by underreporting bias; however, we implemented measures to address this issue, as explained in the Methods section. Additionally, milder cases may be underreported, while more severe cases could potentially be overrepresented. We conducted comprehensive follow-up assessments of patients with severe SAEs to minimize potential bias from participant attrition.

## Conclusion

In conclusion, our study revealed the following important insights: (1) in patients with severe SAEs, heterologous viral vector-primed mRNA vaccination may be associated with a higher risk of mortality compared to homologous viral vector prime-boost vaccination; (2) among patients with severe SAEs, those vaccinated at vaccination centers were associated with better survival outcomes; and (3) heterologous viral vector-primed mRNA vaccination demonstrated increased severe SAEs attributed to respiratory and genitourinary diseases, as well as increased deaths associated with genitourinary diseases in this patient population. Our study, while limited to associations and not establishing causality, provides critical insights for decision-making when heterologous vaccination is needed, such as during vaccine shortages, to manage severe SAEs and improve patient outcomes.

## Supporting information

S1 Text Clinical diagnoses using the international classification of diseases 10th revision (ICD-10) and ICD-10 clinical modification classification schemes, grouping them into specific etiological categories. (DOCX)
