# Supplementary material for: Mortality in severe serious adverse events following heterologous and homologous prime-boost vaccination strategies for SARS-CoV-2: A retrospective cohort study
Source: PLoS One. 2025 May 23;20(5):e0323736. doi: 10.1371/journal.pone.0323736 (PMC12101841; doi:10.1371/journal.pone.0323736)
Supplement: S1 Text — (DOCX) [file pone.0323736.s001.docx]

**S1 Text. Clinical diagnoses using the International Classification of Diseases 10th Revision (ICD-10) and ICD-10 Clinical Modification classification schemes, grouping them into specific etiological categories.**

Certain infectious and parasitic diseases (A00–B99, U07.1, and U07.2); neoplasms (C00–D48); diseases of the blood and blood-forming organs and certain disorders involving the immune mechanism (D50–D89); endocrine, nutritional, and metabolic diseases (E00–E88); mental and behavioral disorders (F00-F99); diseases of the nervous system (G00–G98); diseases of the circulatory system (I00–I99); diseases of the respiratory system (J00–J98 and U04); diseases of the digestive system (K00–K92); diseases of the musculoskeletal and connective tissue (M00–M99); diseases of the genitourinary system (N00–N98); symptoms and signs not classified elsewhere (R00–R99); injury, poisoning, and other consequences of external causes (S00–T98); and external causes of morbidity and mortality (V01–Y98).
